# Evaluation of a novel flexible, lightweight blanket-like torso coil in pediatric MRI at 1.5-T – initial experience

**DOI:** 10.1007/s00247-026-06761-5

**Published:** 2026-08-22

**Authors:** Jonathan R. Dillman, Jean A. Tkach, R. Paul Guillerman, Murat Kocaoglu, Yinan Li, Michael P. Nasser, Adam F. Prasanphanich, Amol Pednekar, Ashan Kharzai, Joshua Greer, Jonathan A. Dudley

**Affiliations:** 1 Department of Radiology, Cincinnati Children’s Hospital Medical Center, 3333 Burnet Avenue, Cincinnati, OH 45229 United States; 2 Department of Radiology, University of Cincinnati, Cincinnati, United States; 3 Philips Healthcare, Best, Netherlands

**Keywords:** Children, Contrast-to-noise ratio, Image quality, MRI, Phased-array coil, Signal-to-noise ratio

## Abstract

**Background:**

Conventional phased-array torso coils used for abdominal MRI are relatively heavy and inflexible, potentially affecting patient comfort and technologist workflow.

**Objective:**

To compare image quality, patient experience, and technologist experience using a recently United States Food and Drug Administration-cleared flexible, lightweight, blanket-like torso coil versus a conventional torso coil for pediatric abdominal MRI.

**Materials and methods:**

In this prospective institutional review board-approved study, 20 pediatric patients (7 years-old to 18 years-old) undergoing routine clinical outpatient abdominal MRI were imaged on the same 1.5-T system using both a conventional torso coil and a flexible blanket-like torso coil (Smart Fit TorsoCardiac; Philips Healthcare). After standard clinical imaging, selected sequences were repeated following coil exchange. Five pediatric radiologists independently evaluated paired image sets for subjective signal-to-noise ratio (SNR) and coil-related artifacts. Objective image quality was assessed using organ- and tissue-based SNR estimates and contrast-to-noise ratio (CNR) measurements. Patients and MRI technologists completed questionnaires regarding comfort, usability, and examination experience.

**Results:**

All 20 patients (median age, 15 years; 55% female) underwent imaging with both coils. Radiologists rated the novel coil as similar to the conventional coil for subjective SNR across all three sequences under study; coil-related artifacts were uncommon (2% of assessments for all sequences evaluated). Objective analysis showed no significant image-based SNR differences for most sequences, although the flexible coil demonstrated higher liver SNR on axial diffusion-weighted imaging (adjusted *P*=0.01). CNR did not differ between coils. Patients reported greater comfort and less anxiety with the flexible coil; 55% preferred it for future MRI examinations versus 20% for the conventional coil. Technologists also favored the flexible coil, with 70% expressing an overall preference.

**Conclusion:**

The flexible blanket-like torso coil provided image quality similar to a conventional coil and was generally preferred by patients and MRI technologists.

## Introduction

Magnetic resonance imaging (MRI) is an essential diagnostic imaging modality in children that provides excellent soft-tissue contrast without the use of ionizing radiation. Continuous advancements in MRI hardware and software have improved image quality, reduced acquisition times, and expanded clinical applications [1–3]. Among these advances, receiver coil technology plays a central role in determining signal-to-noise ratio (SNR), image uniformity, spatial resolution, and accelerated imaging performance [1, 4]. Consequently, innovations in coil design have the potential to significantly impact both image quality and patient experience.

Conventional rigid phased-array torso coils have long been the clinical standard for body MRI examinations. While conventional designs offer robust imaging performance across a wide range of clinical applications, their relatively rigid construction and weight may limit their ability to conform closely to individual patient anatomy. Although coil bridge devices can improve patient comfort by elevating the coil above the body and reducing direct pressure, this creates a gap between the coil and the anatomy of interest. Increasing the distance between the receiver elements and the target anatomy may reduce signal sensitivity and potentially degrade image quality.

Recent developments in flexible radiofrequency (RF) receiver coil technology aim to address these limitations through lightweight, anatomically conformable designs. Flexible coil arrays that drape over the body area of interest can be positioned closer to the patient, potentially improving SNR while enhancing comfort and workflow [5, 6]. Such designs may be especially advantageous in pediatric patients with diverse body habitus and in examinations where optimal coil positioning is challenging [5–7]. The ability of flexible coil technology to improve anatomical conformity while maintaining high-channel-count performance represents a potentially important advancement in MRI hardware. However, the clinical performance of such novel designs relative to established standard-of-care torso coils should be objectively evaluated.

The purpose of this study was to compare the same-scanner, same-patient imaging performance of a recently United States (U.S.) Food and Drug Administration (FDA)-cleared flexible, lightweight blanket-like torso coil with that of a standard conventional coil for pediatric abdominal MRI. Specifically, we evaluated both subjective and objective image quality as well as patient and technologist experiences and preferences. We hypothesized that the novel coil would provide similar imaging performance while improving patient comfort and coil positioning compared with the conventional torso coil.

## Methods

### Study design and participants

Twenty pediatric patients (7 years-old to 18 years-old) undergoing clinically indicated outpatient, non-sedated abdominal MRI examinations were prospectively recruited. The study cohort included 10 patients undergoing routine abdominal MRI for oncologic indications, and 10 patients undergoing magnetic resonance enterography (MRE).

All participants first completed their standard-of-care clinical MRI examinations using a conventional phased-array torso coil. The coil was then exchanged for the novel blanket-like torso coil, after which a localizer and selected study-specific sequences were repeated using identical acquisition parameters and acceleration factors. All imaging was performed during a single session on the same 1.5-T MRI scanner (Ingenia Ambition X; Philips Healthcare, Best, Netherlands). Spine coils (12-elements) embedded within the table of the MRI scanner were similarly active when scanning with both the conventional and novel surface coils.

For the 10 oncologic abdominal MRI examinations, repeated sequences using identical acquisition parameters included: coronal T2-weighted single-shot fast spin echo (SSFSE), axial T2-weighted MultiVane XD (MVXD) fat-suppressed, and axial diffusion-weighted imaging (DWI; highest *b*=800 s/mm^2^). For the 10 MRE examinations, the repeated sequences included coronal T2-weighted SSFSE and axial DWI (highest *b*=800 s/mm^2^). The coronal T2-weighted SSFSE and axial DWI sequences were nearly identical between the oncologic abdominal MRI and MRE protocols with the exception of acquisition acceleration techniques. Across the study cohort, repeated acquisitions included 19 coronal T2-weighted SSFSE, 20 axial DWI, and 10 axial T2-weighted MVXD fat-suppressed sequences (note, repeat coronal T2-weighted SSFSE images were inadvertently not acquired in a single participant). Detailed pulse sequence parameters are provided in Table 1.

**Table 1:** MRI pulse sequence details. For each participant, the exact same acquisition parameters were used for both the novel flexible, lightweight blanket-like, and conventional torso coils

	**Coronal T2W SSFSE**	**Axial T2W MVXD with fat-suppression**	**Axial diffusion-weighted imaging**
Echo time (ms)	80	80	62
Repetition time (ms)	Shortest	Shortest	Shortest
Excitation/refocusing flip angles (degrees)	90/135	90/120	90/180
Number of signal averages	1	1	2/2/4
Type of fat-suppression	NA	SPIR	SPIR
Acceleration	SENSE factor 3 or compressed SENSE factor 3.5	SENSE factor 2 or compressed SENSE factor 2	SENSE factor 2 or compressed SENSE factor 1.8
Respiratory triggering	Variable, including breath-held, free-breathing, and respiratory triggered exams	Yes	Yes
b-values (s/mm^2^)	NA	NA	0/100/800

*DWI*, diffusion-weighted imaging; *FS*, fat-suppressed; *MVXD*, MultiVane XD; *NA*, not applicable; *SENSE*, sensitivity encoding; *SPIR*, spectral presaturation with inversion recovery; *SSFSE*, single-shot fast spin-echo; *T2W*, T2-weighted

### MRI coil details

The novel Smart Fit TorsoCardiac coil (Philips Healthcare, Best, Netherlands) is a flexible, lightweight blanket-like RF phased-array receiver coil that received U.S. FDA clearance in April 2026. The coil weighs 1.8 kg and consists of a 16-element array arranged in a 4×4 configuration within a 55×60 cm (21.6×23.6 inches) footprint, and is constructed using a flexible printed circuit board enclosed within a padded cover. The design permits greater than 90° flexibility in all directions, allowing close conformity to patient anatomy. Low-noise preamplifiers are integrated within the coil housing to improve signal-to-noise ratio (SNR), while receiver and digitization electronics are positioned externally to enhance flexibility and minimize patient burden.

The standard-of-care coil was the dStream Torso coil (Philips Healthcare). The coil weighs 5.1 kg and consists of a 16-element array arranged in a 4×4 configuration within a 55×70 cm (21.6×27.5 inches) footprint.

**Fig. 1:**
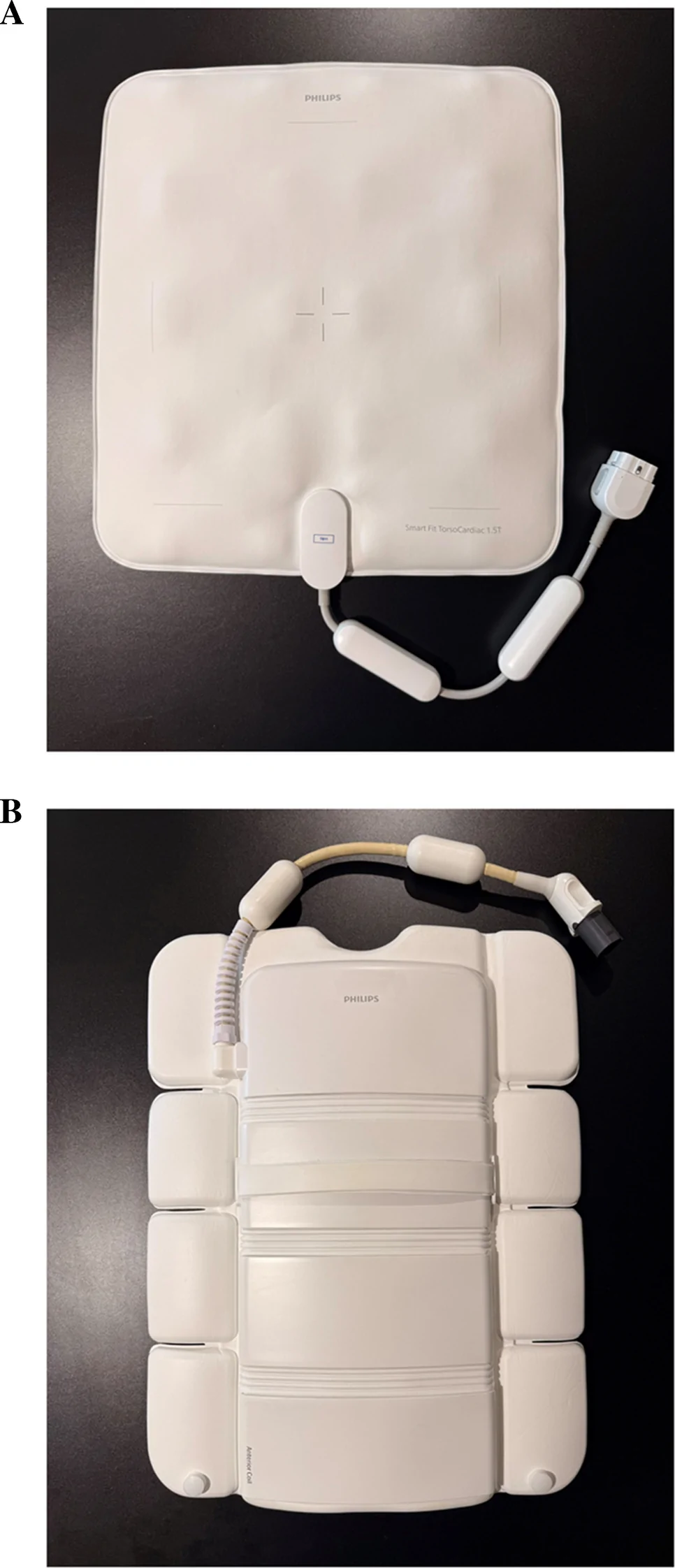
Photographs of the (**a**) novel flexible, lightweight blanket-like (Smart Fit TorsoCardiac) and (**b**) conventional (dStream Torso) phased-array torso coils used in this study

Both coils are presented in Fig. 1.

### Image analysis

#### Subjective radiologist image assessment

Five fellowship-trained pediatric radiologists independently reviewed paired image sets (all images within each set were evaluated) obtained with the conventional and novel coils, with radiologists blinded to one another. Readers assessed subjective SNR using a 3-point comparative scale, where 0 indicated novel coil performance worse than the conventional coil, 1 indicated similar performance, and 2 indicated better performance with the novel coil. Readers also recorded the presence of non-motion-related image artifacts on Smart Fit images that were not present on corresponding conventional coil images, such focal bright or dark areas, ghosting artifacts, and image non-uniformity. For DWI assessments, study radiologists were asked to review only the high *b*-value (800 s/mm^2^) images. Image sets could not be effectively blinded with respect to coil because images acquired with the novel coil were obtained after intravenous contrast material administration, resulting in a darker appearance of the kidneys due to concentrated contrast material within the renal parenchyma and collecting systems.

#### Objective SNR assessment

**Fig. 2:**
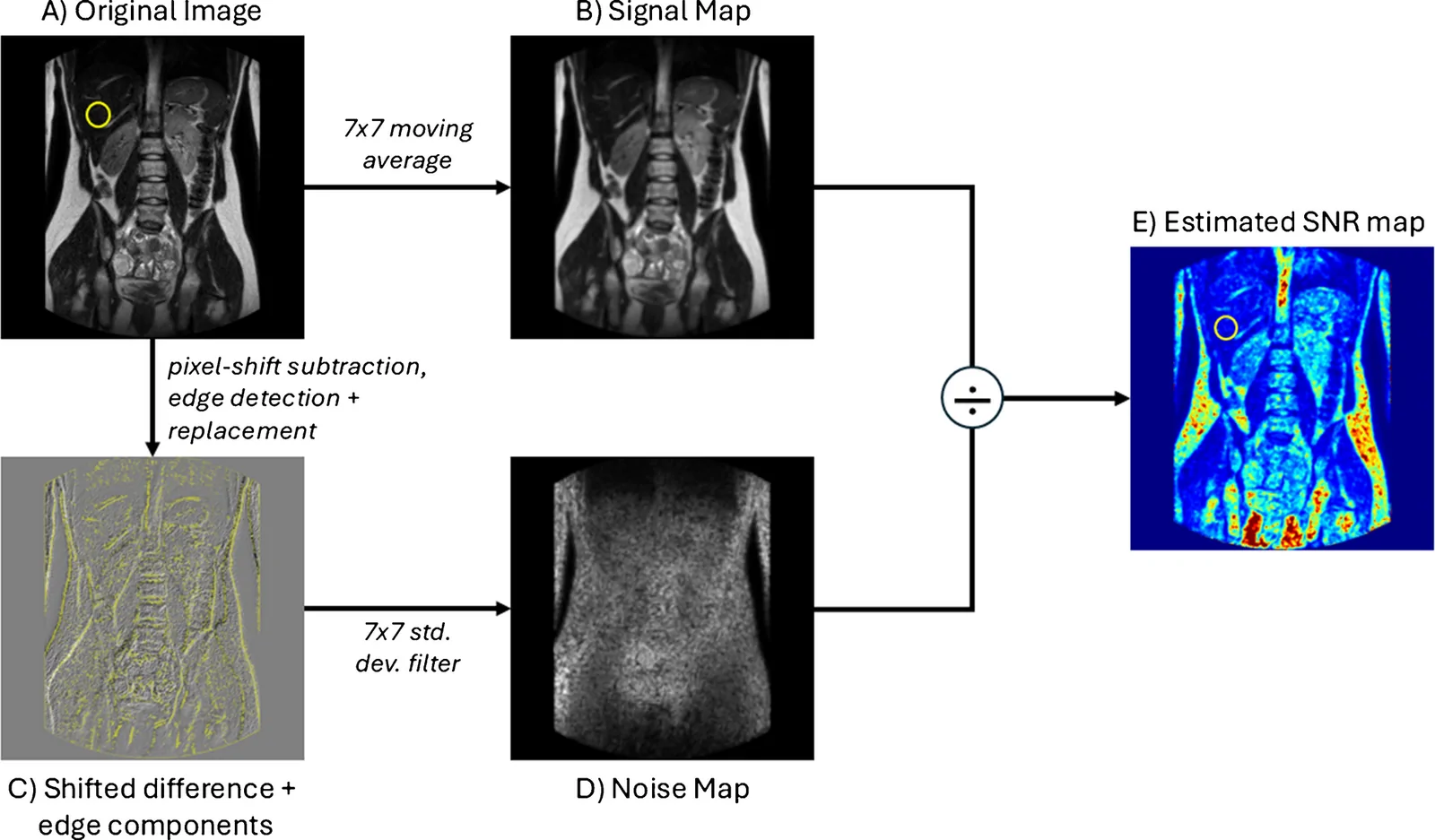
The process of estimating a signal-to-noise ratio (SNR) map for quantitative measurement. The original image (**a**) is first passed through a 7×7 moving average filter to generate a signal map (**b**). Then, a copy of the original image is shifted 1 pixel diagonally and subtracted from the original image; edge components are detected and replaced random noise sampled from a Gaussian model using the local mean and standard deviation ((**c**) shows the subtraction map with detected edge components highlighted in *yellow*). A 7×7 standard deviation filter is applied and the filtered image is divided by the square root of two to generate the noise map (**d**). The estimated SNR map (**e**) is simply the element-wise division of the signal map by the noise map; ROIs from the original image are then applied to the SNR map and the average voxel value within is taken as the image-based SNR estimates

Because dedicated SNR mapping acquisitions were not performed (e.g., repeated acquisitions required for subtraction-map methods), we employed a recently described pixel-shifting method [8] to approximate SNR maps for both conventional and novel coil images. Figure 2 illustrates how image-based SNR estimates were determined. Signal maps were generated from each image by applying a 7×7 pixel moving average filter to calculate the local average signal for each pixel. Then, the image was subtracted by a copy of itself shifted 1 pixel in the phase-encoding direction and 1 pixel in the frequency-encoding direction; from this difference image, edge components were detected and replaced with random noise sampled from a Gaussian model using the local mean and standard deviation of non-edge components. Noise maps were then calculated by computing the standard deviation of the 7×7 pixel neighborhood around every voxel and dividing by the square root of 2. Estimated SNR maps were then obtained by the element-wise division of the signal maps by the noise maps.

For coronal SSFSE and axial T2-weighted MVXD fat-suppressed sequences we used segmentations generated by MRSegmentator model [9] as regions-of-interest (ROI), including for the liver, spleen, pancreas, right kidney, and left kidney as well as the visualized spine, right psoas muscle, and left psoas muscle. Because the MRSegmentator algorithm did not produce accurate segmentations when using axial DWI images as an input, three manually drawn ROIs were used (see “Objective contrast-to-noise ratio assessment” below for details). Image-based SNR estimates for each ROI were calculated as the mean value from the voxels of the estimated SNR map within the respective ROI.

#### Objective contrast-to-noise ratio assessment

We also computed a Contrast-to-Noise Ratio (CNR) which was defined as the absolute difference between mean signal intensities of an ROI of organ of interest and an ROI of a reference muscle divided by the square root of the pooled variance of these ROIs:

$$\mathrm{C}\mathrm{N}\mathrm{R}= \frac{\left|{\mu}_{\mathrm{o}\mathrm{r}\mathrm{g}\mathrm{a}\mathrm{n}}-{\mu}_{\mathrm{m}\mathrm{u}\mathrm{s}\mathrm{c}\mathrm{l}\mathrm{e}}\right|}{\sqrt{{\sigma}_{\mathrm{o}\mathrm{r}\mathrm{g}\mathrm{a}\mathrm{n}}^{2}+{\sigma}_{\mathrm{m}\mathrm{u}\mathrm{s}\mathrm{c}\mathrm{l}\mathrm{e}}^{2}}}$$

For non-diffusion-weighted sequences, five three-dimensional spherical ROIs were manually placed within the liver, spleen, pancreas, and bilateral psoas muscles on conventional coil images. For diffusion-weighted images, three spherical ROIs were placed within the liver, spleen, and left paraspinal muscle on the conventional coil images (the pancreas was not selected due to inconsistent visualization). All ROIs were drawn using 3D Slicer (version 5.8.0) [10]; diameters ranged from 12–30 mm and were selected as the largest allowable size for each patient’s anatomy. To ensure ROIs encompassed the same anatomy for each pair of images, ROIs were transferred from conventional coil images to novel coil images using a two-stage registration process. First, a global three-dimensional rigid registration was performed between the conventional and novel coil image volumes. Second, to account for localized non-rigid motion (e.g., bowel peristalsis), each ROI-containing slice was masked to include voxels within 25 mm of the ROI and underwent local two-dimensional rigid registration. The resulting global and local transformation matrices were combined and applied to the original ROI coordinates to generate corresponding ROIs on the novel coil images.

### Patient and technologist assessment

Following image acquisition, study participants (i.e., patients) and MRI technologists completed separate questionnaires evaluating coil usability, comfort, and overall examination experience. Survey questions are summarized in Table 2.

**Table 2:** Patient and MRI technologist questionnaire questions evaluating MRI coil usability, comfort, and overall examination experience

Survey participant	Question	Possible responses
**Patient**	1. Overall, how comfortable was the first (conventional) surface coil?	Very comfortable Somewhat comfortable Neutral Somewhat uncomfortable Very uncomfortable
	2. Overall, how comfortable was the second (Smart Fit) surface coil?	Very comfortable Somewhat comfortable Neutral Somewhat uncomfortable Very uncomfortable
	3. Did the first (conventional) surface coil contribute to any feelings of anxiety or claustrophobia?	Not at all A little A moderate amount A lot Extremely
	4. Did the second (Smart Fit) surface coil contribute to any feelings of anxiety or claustrophobia?	Not at all A little A moderate amount A lot Extremely
	5. If you needed another MRI scan in the future, which surface coil would you prefer?	First (conventional) Second (Smart Fit) No preference
**MRI technologist**	6. Which surface coil fit your patient’s anatomy better/stayed in place better?	First (conventional) Second (Smart Fit) Similar
	7. Which surface coil was easier to position and secure on the patient?	First (conventional) Second (Smart Fit) Similar
	8. Which surface coil more positively impacted workflow?	First (conventional) Second (Smart Fit) Similar
	9. If your patient had to undergo another MRI exam in the future, which coil would you use?	First (conventional) Second (Smart Fit) No preference

### Statistical analysis

Continuous variables were summarized using medians and interquartile ranges. Categorical variables were summarized using counts and percentages. Subjective radiologist assessments and survey responses were reported descriptively. Objective image-based SNR and CNR measurements obtained with the novel and conventional coils were compared using the nonparametric Wilcoxon signed-rank test. To account for multiple comparisons, *P*-values were adjusted using the Benjamini–Hochberg false discovery rate (FDR) procedure [11]. Statistical significance was defined as a two-sided adjusted *P*-value <0.05.

## Results

### Participants

A total of 20 participants were included in the study. The cohort comprised 11 females (55%) and 9 males (45%). The median age of the participants was 15 years, with ages ranging from 7 years to 18 years.

### Radiologist assessments

Five radiologist readers independently assessed subjective SNR and the presence of artifacts on images acquired using the novel coil compared with the conventional coil. Across all sequence types and for all radiologists combined, the novel coil was rated as similar to the conventional coil based on perceived SNR (Table 3). Median SNR score was 1 for all three sequences assessed, indicating there was no difference between the novel coil and standard-of-care imaging for the majority of patients.

**Table 3:** Subjective human radiologist median assessments performed by five radiologists presented by reader and pulse sequence. Readers assessed subjective SNR using a 3-point comparative scale, where 0 indicated novel (Smart Fit) performance worse than the conventional coil, 1 indicated similar performance, and 2 indicated better performance with the novel (Smart Fit) coil. Interquartile ranges are presented in parentheses. Readers also recorded the presence of image artifacts on novel (Smart Fit) images that were not present on corresponding conventional coil images

	Coronal SSFSE (***n***=19)		Axial T2W MVXD FS (***n***=10)		Axial DWI (***n***=20)	
	SNR score	Artifacts (%)	SNR score	Artifacts (%)	SNR score	Artifacts (%)
**Reader 1**	1 (1, 1)	0	1 (1, 2)	0	1 (1, 1)	0
**Reader 2**	2 (2, 2)	5	2 (2, 2)	10	1 (1, 1)	10
**Reader 3**	0 (0, 1)	5	1 (0.25, 1)	0	1 (0.75, 1)	0
**Reader 4**	1 (1, 1)	0	1 (1, 2)	0	1 (1, 10	0
**Reader 5**	1 (1, 1)	0	1 (1, 2)	0	1 (1, 1)	0
**Median**	1	2	1	2	1	2

*DWI*, diffusion-weighted imaging; *FS*, fat-suppressed; *MVXD*, MultiVane XD; *SNR*, signal-to-noise ratio; *SSFSE*, single-shot fast spin-echo; *T2W*, T2-weighted

Artifacts unique to images obtained using the novel coil were uncommon, documented on 2% of coronal SSFSE, 2% of axial T2W MVXD fat-suppressed, and 2% of axial DWI assessments (Table 3).

### Objective SNR assessment

**Fig. 3:**
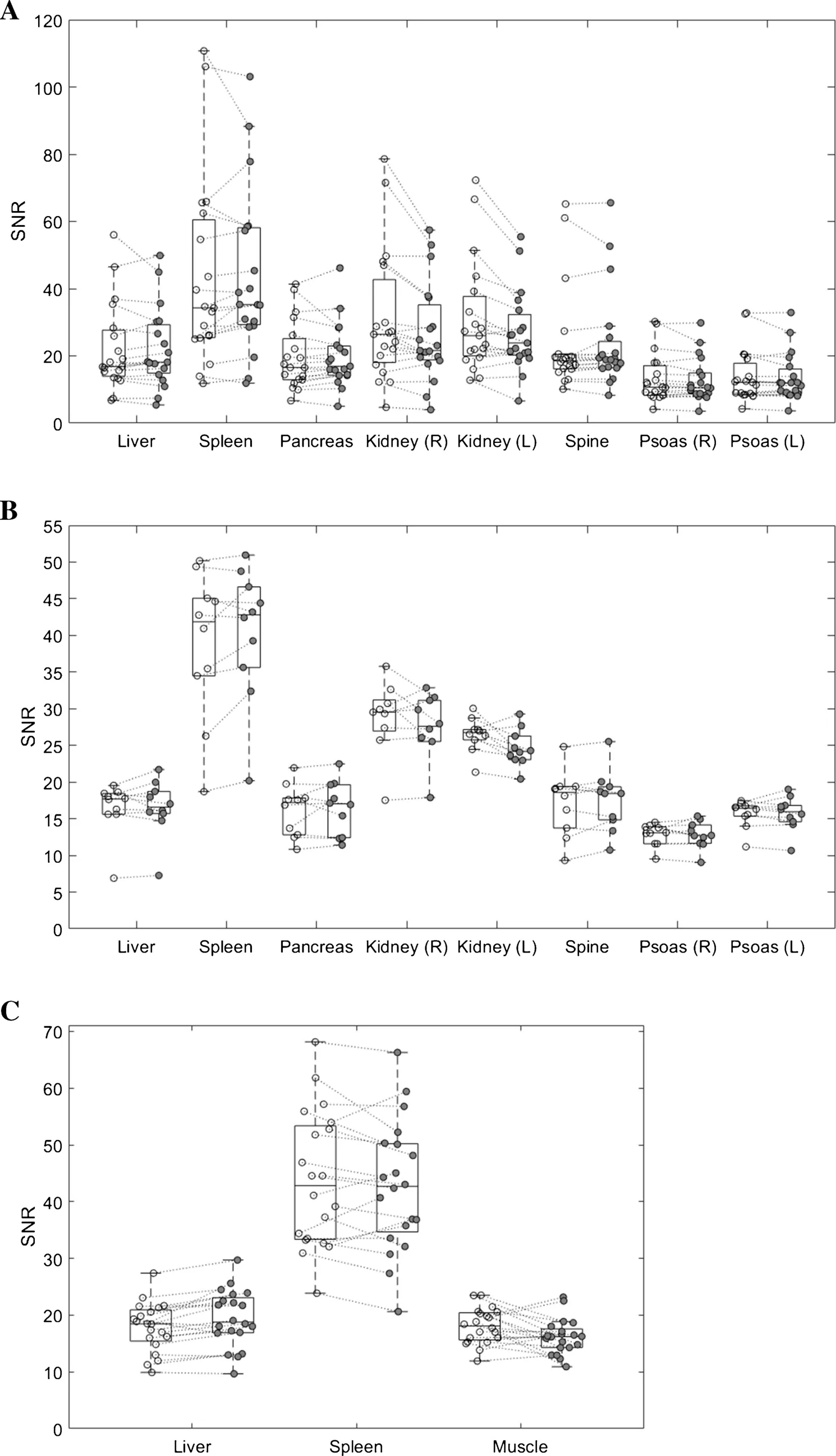
Tukey box plots with connecting lines showing estimated image-based SNR by organ or tissue for the novel flexible, lightweight blanket-like (Smart Fit; *gray circles*) and conventional (*white circles*) phased-array torso coils used in this study. **a** Coronal T2-weighted single-shot fast spin-echo sequence. **b** Axial T2-weighted MultiVane XD fat-suppressed sequence. **c** Axial diffusion-weighted imaging (*b*=800 s/mm^2^)

There were no significant differences in objective image-based SNR estimates between the two coils examined for any organ or tissue evaluated on coronal SSFSE or axial T2-weighted MVXD fat-suppressed images. On axial DWI, the novel coil demonstrated significantly higher SNR in the liver (p-FDR=0.01) compared to the conventional coil. No significant differences in SNR were observed between coils for the spleen or muscle on axial DWI images. SNR estimates, uncorrected *P*-values, and FDR-corrected *P*-values are summarized in Table 4, while SNR estimates are shown on Fig. 3.

**Table 4:** Median and interquartile range of image-based SNR estimates by organ or tissue for the conventional and novel (Smart Fit) coils. *P*-values shown are from a two-tailed nonparametric Wilcoxon signed-rank test, along with the false-discovery rate corrected *P*-values (p-FDR). Note, one participant did not have a right kidney

	**Organ/tissue**	**Conventional**	**Smart Fit**	***P*-value**	*p*-FDR
**Coronal T2W SSFSE** **(*n *= 19)**	Liver	16.84 (13.97–27.68)	18.13 (14.88–29.27)	0.546	0.546
	Spleen	34.29 (25.46–60.49)	35.29 (29.29–58.11)	0.260	0.416
	Pancreas	16.53 (12.91–25.13)	16.91 (14.24–22.96)	0.376	0.430
	Kidney (R)	26.43 (18.05–42.70)	21.58 (18.84–35.19)	0.013	0.063
	Kidney (L)	26.02 (20.02–37.69)	23.83 (20.22–32.31)	0.016	0.063
	Spine	18.53 (16.13–20.41)	18.90 (16.47–24.30)	0.091	0.243
	Psoas (R)	10.71 (8.25–17.10)	10.52 (8.72–14.95)	0.159	0.318
	Psoas (L)	12.11 (8.56–17.83)	11.90 (8.81–16.09)	0.376	0.430
**Axial T2W MVXD FS** **(*n *= 10)**	Liver	17.69 (15.61–18.45)	16.58 (15.72–18.72)	0.922	0.922
	Spleen	41.85 (34.49–45.08)	42.81 (35.63–46.62)	0.160	0.641
	Pancreas	17.21 (12.81–17.83)	17.03 (12.41–19.64)	0.625	0.922
	Kidney (R)	29.54 (26.97–31.20)	27.60 (25.53–31.15)	0.820	0.922
	Kidney (L)	26.75 (25.75–27.17)	24.17 (23.05–26.27)	0.037	0.297
	Spine	18.59 (13.74–19.40)	18.45 (14.85–19.37)	0.375	0.922
	Psoas (R)	13.07 (11.59–13.89)	12.71 (11.66–14.15)	0.846	0.922
	Psoas (L)	16.42 (15.36–16.77)	15.95 (14.60–16.82)	0.695	0.922
**Axial DWI (*n *= 20)**	Liver	18.45 (15.42–20.93)	18.76 (16.89–23.07)	0.003	0.010
	Spleen	42.82 (33.36–53.36)	42.70 (34.66–50.19)	0.263	0.263
	Muscle	18.07 (15.63–20.42)	16.21 (14.28–17.57)	0.052	0.078

*DWI*, diffusion-weighted imaging; *FS*, fat-suppressed; *MVXD*, MultiVane XD; *SSFSE*, single-shot fast spin-echo; *T2W*, T2-weighted

### Objective CNR assessment

**Fig. 4:**
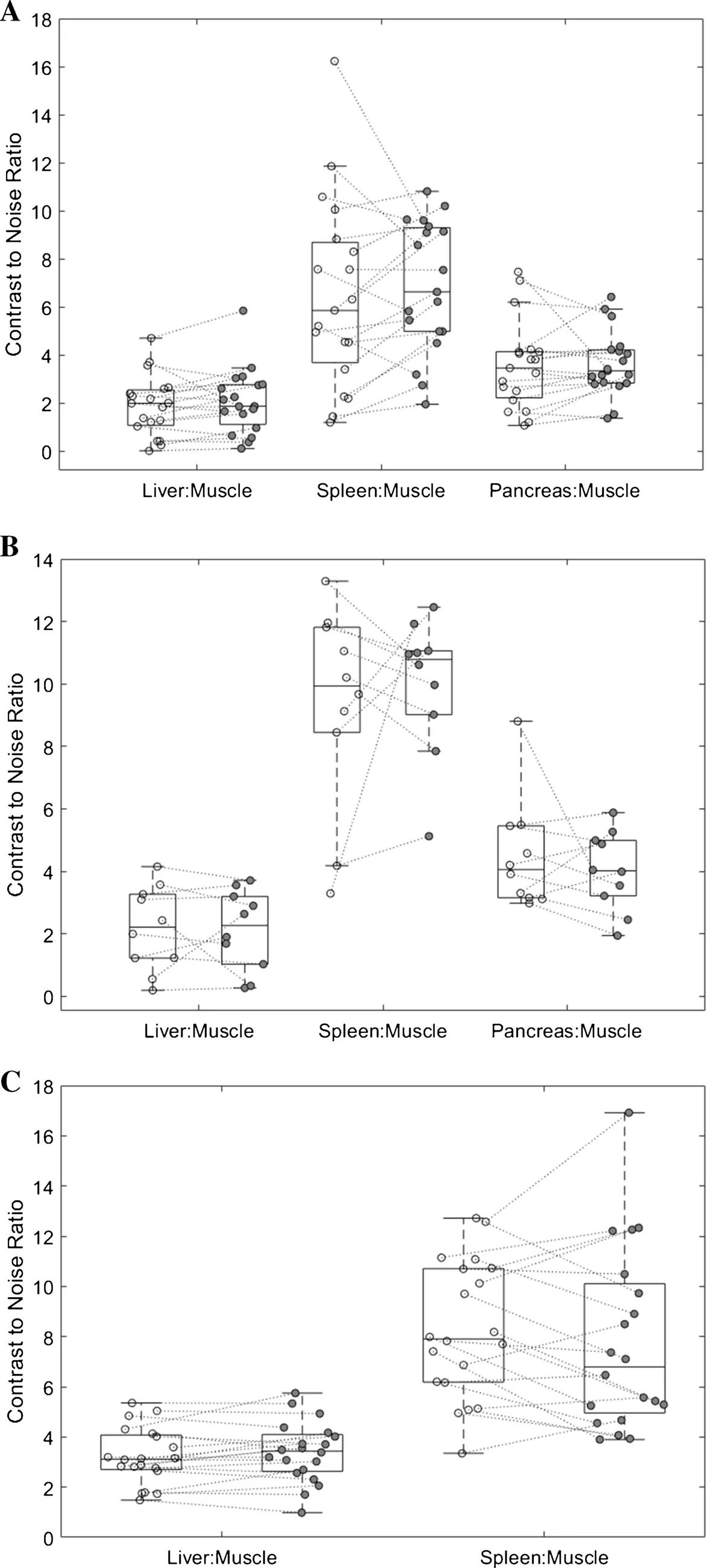
Tukey box plots with connecting lines showing estimated CNR by organ or tissue for the novel flexible, lightweight blanket-like (Smart Fit; *gray circles*) and conventional (*white circles*) phased-array torso coils used in this study. **a** Coronal T2-weighted single-shot fast spin-echo sequence. **b** Axial T2-weighted MultiVane XD fat-suppressed sequence. **c** Axial diffusion-weighted imaging (*b*=800 s/mm^2^)

There were no significant differences in CNR between the two coils for any organ or tissue for any sequence. CNR estimates, uncorrected *P*-values, and FDR-corrected *P*-values are summarized in Table 5, while CNR estimates are shown in Fig. 4.

**Table 5:** Median and interquartile range of CNR by organ or tissue for the conventional versus novel (Smart Fit) coils. *P*-values shown are from a two-tailed nonparametric Wilcoxon signed-rank test, along with the false-discovery rate corrected *P*-values (p-FDR)

	**Organ/tissue**	**Conventional**	**Smart Fit**	***P*-value**	*p*-FDR
**Coronal T2W SSFSE** **(*n*=19)**	Liver	1.99 (1.08–2.55)	1.87 (1.11–2.77)	0.126	0.341
	Spleen	5.86 (3.69–8.69)	6.63 (4.98–9.30)	0.227	0.341
	Pancreas	3.46 (2.22–4.14)	3.34 (2.84–4.21)	0.400	0.400
**Axial T2W MVXD FS** **(*n*=10)**	Liver	2.21 (1.22–3.37)	2.27 (1.03–3.20)	0.695	0.922
	Spleen	9.94 (8.45–11.81)	10.78 (9.02–11.06)	0.922	0.922
	Pancreas	4.06 (3.15–5.46)	4.02 (3.22–4.99)	0.492	0.922
**Axial DWI (*n*=20)**	Liver	3.11 (2.70–4.07)	3.43 (2.63–4.09)	0.370	0.370
	Spleen	7.90 (6.18–10.71)	6.79 (4.96–10.11)	0.191	0.370

*DWI*, diffusion-weighted imaging; *FS*, fat-suppressed; *MVXD*, MultiVane XD; *SSFSE*, single-shot fast spin-echo; *T2W*, T2-weighted

### Patient assessment

Twenty participants completed the patient experience questionnaire. Regarding comfort ratings for the conventional coil, 9/20 (45%) participants reported being very comfortable, 3/20 (15%) somewhat comfortable, 4/20 (20%) neutral, and 4/20 (20%) somewhat uncomfortable, with no participants reporting being very uncomfortable. In contrast, for the novel coil, 9/20 (45%) participants reported being very comfortable and 9/20 (45%) somewhat comfortable, while only 1/20 (5%) reported a neutral experience and 1/20 (5%) reported being somewhat uncomfortable; no participants reported being very uncomfortable.

Regarding patient-reported anxiety and claustrophobia for the conventional coil, 10/20 (50%) participants reported that the coil did not contribute to feelings of anxiety or claustrophobia at all, while 7/20 (35%) reported a little contribution and 3/20 (15%) reported a moderate contribution. No participants reported experiencing a lot or extreme anxiety/claustrophobia with the conventional coil. In comparison, 16/20 participants (80%) reported no anxiety or claustrophobia associated with the novel coil, 3/20 (15%) reported a little contribution, and only 1/20 (5%) reported a moderate contribution; no participants reported a lot or extreme anxiety/claustrophobia.

When asked which coil they would prefer for a future MRI examination, 11/20 participants (55%) preferred the novel coil, compared with 4/20 (20%) who preferred the conventional coil, while 5/20 (25%) expressed no preference.

### MRI technologist assessment

When asked which coil better conformed to the patient’s anatomy and remained in position throughout the examination, MRI technologists favored the novel coil in 9/20 (45%) examinations, compared with 1/20 (5%) examinations favoring the conventional coil; in the remaining 10 (50%) examinations, the coils were rated as similar. Identical results were observed for ease of positioning and securing the coil on the patient, with technologists favoring the novel coil in 45% of examinations, the conventional coil in 5%, and reporting no difference in 50%. Regarding workflow, the novel coil was considered to have a more positive impact in 8/20 (40%) examinations, while no examinations favored the conventional coil and 12/20 (60%) were rated as having similar workflow performance. When asked which coil they would choose for a future MRI examination, technologists selected the novel coil in 14/20 (70%) cases compared with 1/20 (5%) cases for the conventional coil, while indicating no preference in 5/20 (25%) cases. Assessments were completed by eight unique highly experienced pediatric technologists, with 12 years to 29 years of experience in MRI.

**Fig. 5:**
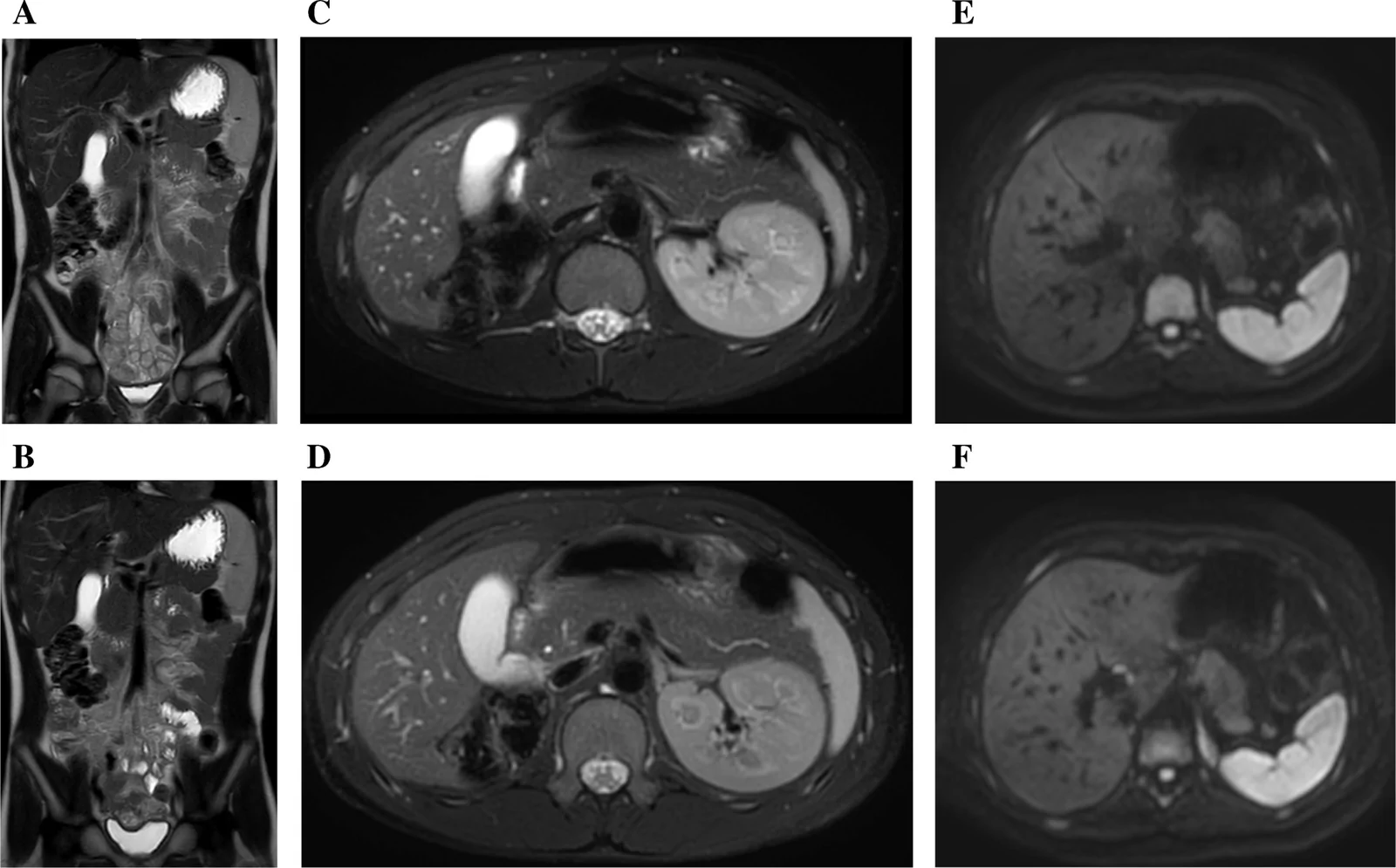
Example images obtained using two different phased-array torso coils at 1.5 T from an 8-year-old boy. **a**, **b** Coronal T2-weighted single-shot fast spin-echo MR images using conventional and novel flexible, lightweight blanket-like surface coils. **c**, **d** Axial T2-weighted MultiVane XD fat-suppressed images using conventional and novel surface coils. **e**, **f** Axial diffusion-weighted images (*b*=800 s/mm^2^) using conventional and novel surface coils. Overall reader (*n*=5) median perceived signal-to-noise ratio scores were 1.0 for all three sequences assessed

Examples of images obtained using both coils in the same participant are shown in Fig. 5.

## Discussion

In this prospective same-patient, same-scanner comparison of a recently U.S. FDA-cleared flexible, lightweight blanket-like phased-array torso coil and a conventional torso coil for pediatric abdominal MRI at 1.5-T, we found that the novel coil provided imaging performance comparable to standard-of-care imaging while offering several advantages related to patient and technologist experience. Across multiple abdominal MRI pulse sequences, objective measures of image quality showed no consistent differences between coil configurations, while subjective radiologist assessments suggested no difference in perceived SNR. Importantly, the novel coil did not introduce clinically meaningful additional image artifacts compared to the conventional coil. Beyond imaging performance, both patients and MRI technologists frequently preferred the novel coil when a preference was expressed, supporting its potential to improve the overall MRI examination experience without compromising diagnostic image quality.

Objective image quality analysis demonstrated no meaningful differences between the novel and conventional coils. Across coronal T2-weighted SSFSE and axial T2-weighted MVXD fat-suppressed sequences, image-based SNR measurements were similar for all evaluated organs and tissues without statistically significant differences. Although higher liver SNR was observed with the novel coil on axial DWI images after correction for multiple comparisons (perhaps due to differences in anterior surface coil geometries and sensitivity profiles), this isolated observation was not accompanied by consistent differences across other tissues or sequences. Similarly, no significant differences in CNR were identified for any organ or tissue across any sequence type. Taken together, these findings suggest that the novel coil likely achieves very comparable objective image quality to that of the conventional coil, supporting the postulate that improvements in patient comfort and workflow can be achieved without sacrificing diagnostic performance.

Subjective radiologist assessments further support the clinical viability of the novel coil. Across all reader and sequence evaluations (*n*=15), the novel coil was rated as similar to or better than the conventional coil with respect to perceived SNR in 14 instances. Only one of the 15 perceived SNR assessments favored the conventional coil (coronal SSFSE imaging for a single reader); the reason for this discrepancy is unclear, as the same reader rated the novel coil as having similar or higher perceived SNR for the other two pulse sequences evaluated. In addition, artifacts unique to the novel coil were very rare, and were not observed by most readers, occurring in only 2% of evaluations for each sequence type. This low artifact burden supports the seamless integration of the flexible coil into routine pediatric abdominal MRI workflows.

Not unexpectedly, patient and technologist feedback revealed some of the most compelling advantages of the novel blanket-like coil. Patients descriptively reported greater comfort and less anxiety or claustrophobia when imaged with the flexible, lighter coil. When asked about future imaging preferences, more than half of participants selected the novel coil, whereas only one-fifth preferred the conventional coil. MRI technologists similarly favored the novel coil, particularly with respect to anatomic conformity, coil positioning, and maintaining stable placement throughout the imaging session. Again, whenever a preference between coils was expressed by technologists for future imaging, it was overwhelmingly in favor of the novel coil suggesting workflow advantages.

Prior studies have also demonstrated the feasibility and performance of novel flexible torso MRI coils in pediatric imaging. Winkler et al. developed a pediatric-sized 12-channel screen-printed flexible coil array and evaluated its performance in 20 children ranging from 2 days to 12 years of age [5]. Compared with a conventional commercial coil, the screen-printed coil demonstrated comparable SNR across multiple abdominal muscle groups, with no statistically significant differences, while maintaining high diagnostic image quality. In addition, caregivers expressed a preference for the flexible coil over the conventional design, highlighting potential improvements in patient comfort and clinical acceptance. Similarly, Lee et al. designed and evaluated a highly flexible small-loop array for 3-T MRI, demonstrating superior parallel imaging performance and improved high-spatial-resolution imaging in both phantom studies and adult and pediatric volunteer imaging compared with commercial reference coils [7]. Together, these studies, along with our results, support the growing evidence that flexible coil technology can provide image quality comparable or superior to conventional rigid coil arrays while offering ergonomic advantages that may be particularly beneficial in pediatric patients.

Our study has limitations. First, the sample size was relatively small and consisted of only 20 pediatric participants from a single institution. However, this limitation is partially mitigated by the study design, which included same-patient, same-scanner comparisons, five independent radiologist readers, comparison of multiple pulse sequences commonly used for routine abdominal MR, and highly consistent findings across subjective, objective, patient-reported, and technologist-reported outcomes. Second, objective SNR measurements were estimated using an indirect methodology rather than dedicated SNR mapping acquisitions; because this approach requires optimization of a tuning parameter using ground-truth SNR maps with similar anatomy and image contrast (which was not feasible in the present study), the accuracy of absolute SNR values is potentially limited and should not be compared to SNR values reported in other studies. Nevertheless, the same methodology was applied to both coil configurations, making relative comparisons between coils appropriate.

Third, although study radiologists were instructed to evaluate images from both coils side by side, effective blinding was not possible because the images acquired with the novel coil were obtained after intravenous contrast material administration. As a result, the kidneys appeared darker due to concentrated contrast material within the renal parenchyma and collecting systems, making the image source readily identifiable. Fourth, because our study used a non-randomized image acquisition order, with images acquired using the conventional coil always obtained first and those acquired using the novel coil obtained later in the examination, the results are subject to potential bias. For example, patients may have had greater difficulty remaining still or holding their breath later in the imaging session, which could have influenced subjective radiologist assessments and patient-reported outcomes. Fifth, it is conceivable that contrast material could impact objective SNR and CNR measurements as gadopiclenol not only affects T1 relaxation, but also causes T2 shortening. While this T2 relaxation effect is expected to be minimal for most organs and tissues (excluding the kidneys and renal collecting systems), it has the potential to reduce SNR and CNR for images obtained with the novel coil; therefore, image quality metrics from the novel coil under investigation may have been slightly underestimated. Finally, the novel coil evaluated in this study is available only for a single MRI vendor platform. Despite this limitation, the blanket-like coil technology in this study has the potential to improve MRI experiences for large numbers of pediatric and adult patients worldwide, while simultaneously benefiting MRI technologists through improved workflow.

In conclusion, a recently developed flexible, lightweight blanket-like phased-array torso coil demonstrates subjective and objective image quality comparable to that of a conventional torso coil for pediatric abdominal MRI. Both patients and MRI technologists express an overall preference for the novel coil for future imaging examinations. Collectively, these findings support the integration of flexible, lightweight coil technology into pediatric abdominal MRI to preserve high-quality imaging while improving the experience for both patients and imaging personnel.

## Data Availability

Data were generated for this study and are available for non-commercial purposes upon reasonable request.

## References

[1] Shang Y, Simegn GL, Gillen K et al (2024) Advancements in MR hardware systems and magnetic field control: B0 shimming, RF coils, and gradient techniques for enhancing magnetic resonance imaging and spectroscopy. Psychoradiology 4:kkae013 10.1093/psyrad/kkae013 PMC11384915 39258223

[2] Sneag DB, Abel F, Potter HG et al (2023) MRI advancements in musculoskeletal clinical and research practice. Radiol 308:e230531 10.1148/radiol.230531 PMC10477516 37581501

[3] Heckel R, Jacob M, Chaudhari A et al (2024) Deep learning for accelerated and robust MRI reconstruction. MAGMA 37:335–368 10.1007/s10334-024-01173-8 PMC11316714 39042206

[4] Gruber B, Froeling M, Leiner T, Klomp DWJ (2018) RF coils: a practical guide for nonphysicists. J Magn Reson Imaging 48:590–604 10.1002/jmri.26187 PMC6175221 29897651

[5] Winkler SA, Corea J, Lechêne B et al (2019) Evaluation of a flexible 12-channel screen-printed pediatric MRI coil. Radiol 291:180–185 10.1148/radiol.2019181883 PMC6444619 30806599

[6] Zhang T, Grafendorfer T, Cheng JY et al (2016) A semiflexible 64-channel receive-only phased array for pediatric body MRI at 3T. Magn Reson Med 76:1015–1021 10.1002/mrm.25999 PMC4811745 26418283

[7] Lee W, Stickle Y, Follante C et al (2025) A 60-channel high-density flexible receive array for pediatric abdominal MRI. Magn Reson Med 93:2655–2666 10.1002/mrm.30456 PMC13005962 39902561

[8] Kojima S, Higuchi S, Hayashi T et al (2025) Practical signal-to-noise ratio mapping using single clinical MR images. Radiol Phys Technol 18:972–987 10.1007/s12194-025-00944-9 40736937

[9] Häntze H, Xu L, Mertens CJ et al (2025) Segmenting whole-body MRI and CT for multiorgan anatomic structure delineation. Radiol Artif Intell 7:e240777 10.1148/ryai.240777 40767616

[10] Fedorov A, Beichel R, Kalpathy-Cramer J et al (2012) 3D Slicer as an image computing platform for the Quantitative Imaging Network. Magn Reson Imaging 30:1323–1341 10.1016/j.mri.2012.05.001 PMC3466397 22770690

[11] Chen SY, Feng Z, Yi X (2017) A general introduction to adjustment for multiple comparisons. J Thorac Dis 9:1725–1729 10.21037/jtd.2017.05.34 PMC5506159 28740688

